# Toxicogenomics to Improve Comprehension of the Mechanisms Underlying Responses of *In Vitro* and *In Vivo* Systems to Nanomaterials: A Review 

**DOI:** 10.2174/138920208786847962

**Published:** 2008-12

**Authors:** Anna Poma, Maria L Di Giorgio

**Affiliations:** Department of Basic and Applied Biology, University of L’Aquila, Via Vetoio 1- 67100, L’Aquila, Italy

**Keywords:** Nanomaterials, toxicogenomics.

## Abstract

Engineered nanomaterials are commonly defined as materials with at least one dimension of 100 nanometers or less. Such materials typically possess nanostructure-dependent properties (e.g., chemical, mechanical, electrical, optical, magnetic, biological), which make them desiderable for commercial or medical application. However, these same properties may potentially lead to nanostructure-dependent biological activity that differs from and is not directly predicted by the bulk properties of the constitutive chemicals and compounds. Nanoparticles and nanomaterials can be on the same scale of living cells components, including proteins, nucleic acids, lipids and cellular organelles. When considering nanoparticles it must be asked how man-made nanostructures can interact with or influence biological systems. Carbon nanotubes (CNTs) are an example of carbon-based nanomaterial, which has won a huge spreading in nanotechnology. The incorporation of CNTs in living systems has raised many concerns because of their hydrophobicity and tendency to aggregate and accumulate into cells, organs, and tissues with dangerous effects.

Applications of toxicogenomics to both investigative and predictive toxicology will contribute to the in-depth investigation of molecular mechanisms or the mode of nanomaterials action that is achieved by using conventional toxicological approaches. Parallel toxicogenomic technologies will promote a valuable platform for the development of biomarkers, in order to predict possible nanomaterial’s toxicity. The potential of characteristic gene expression profiles (“fingerprint”) of exposure or toxicological response to nanoparticles will be discussed in the review to enhance comprehension of the molecular mechanism of *in vivo* and *in vitro* system exposed to nanomaterials.

## INTRODUCTION

Nanomaterial can be defined as a material having structure on a scale greater than atomic/molecular dimensions, but less than 100 nm, which exhibits physical, chemical and biological characteristics associated with its nanostructure. 

There are many application fields for these nanomaterials such as high performance materials, energy storage and conversion, self-cleaning surface coatings and stain-resistant textiles using simple nanostructured materials such as carbon nanotubes and metal oxide nanoparticles.

Research into more complex nanomaterials will lead to applications such as cellular-level medical diagnostics and treatment [1]. Nanotechnology is an emerging field, the benefits of which are widely publicized. Nanomaterials are present in a number of commercially available products including sunscreens, cosmetics and many industrial applications, but there are uncertainties as to whether the unique properties that support their commercial use may also pose potential occupational health risks [2]. Nanomaterials have a high surface-to-volume ratio, so surface reactivity will be high. These particles may adopt structures that are different from the bulk form of the chemical and, therefore, may exhibit different chemical and physical properties [3]. Ultrafine particulate matter is a well-known example of ambient nanoscale particles. Moreover, great attention of the scientific world is being directed to manufactured nanoscale materials of current or projected commercial importance. 

Nanoscale materials are already becoming commercially available for industrial applications and consumer use and in the fields of biology and medicine as drug and delivery formulations, for tissue engineering, for destroying tumors by hyperthermia, for probes of DNA structure, and for biosensors [4, 5, 6]. Uptil now less information is available regarding air-born levels of nanomaterials generated during production or quantities, which may be aerosolized into the environment. 

Ultrafine particle inhalation toxicology studies suggest that particle size can influence toxicity principally due to two factors: the large surface area and its reactivity or intrinsic toxicity [7]. If surface chemistry is influenced by the size of the particle, surface properties can be changed by coating nanoscale particles with different materials. This interaction of surface area and particle composition in eliciting biological responses adds an extra dimension of complexity in evaluating potential adverse events that may result from exposure to these materials [8]. There are indications in the literature that manufactured nanoscale materials may spread in the body in unpredictable ways, and certain nanoscale materials have been observed to preferentially accumulate in particular organelles. Furthermore, the unique and diverse physicochemical properties of nanoscale materials suggest that their toxicological properties may differ from those of the corresponding bulk materials [9].

Biocompatibility, toxicity and the ability to penetrate cells are three critical factors that will determine the utility of nanoparticles in clinical applications. Further, widespread clinical use and inclusion in consumer products require large scale production, which raises concerns about safe exposure to nanomaterials even at low concentrations [10]. However, there is little information on the consequences of nanoparticles exposure to living system.

Whole-body studies show that inhalation of nanoparticles and entry through the lungs is followed by rapid translocation to vital organs, like the kidney and liver [11]. Moreover, nanoparticles toxicity can be attributed to: release of toxic ions, for example CdSe/ZnS nanoparticles, nonspecific interaction with biological structures facilitated by their shape, as in the case of nanotubes [12], and specific interaction with biomacromolecules through surface modifications. Particle (or aggregate) size determines whether a particle enters the cellular environment through phagocytosis, a process of engulfment by which macrophages ingest cellular fragment or micro particles, through endocytosis, or some undefined mechanisms [13, 14]. In a recent work Porter A. *et al. *(2007) [15] showed for the first time, a bundle of single-walled carbon nanotubes (SWCNTs) within the nucleus of human macrophage like treated cells. Uptake to these sites implies that they may interact with intracellular proteins, organelles and DNA, which would greatly enhance their toxic potential. SWNTs are also fused with the plasma membrane, where they have been shown to cause cell damage through lipid peroxidation and oxidative stress [16, 17].

A new approach to understand the molecular mechanism at the base of toxicity induced by nanomaterials is the toxicogenomics, resulting from the merge of conventional toxicology with functional genomics. Toxicogenomics is a scientific field that studies the complex interaction between the structure and activity of the genome and adverse biological effects caused by exogenous agents such as toxins, drugs, and environmental stressors. Nanomaterials distribution to different body tissues following deposition in the respiratory tract or administered to an *in vivo* system can potentially affect multiple cellular functions. It will be difficult to determine with conventional assay what changes and adverse effects may have occurred. Use of genomic approach provides information about specific mechanisms at the molecular level (e.g. oxidative stress); in particular, toxicogenomics has proved to be a powerful tool for the direct monitoring of patterns of cellular pertubations in specific pathways, through identification and quantification of global shifts in gene expression resulting within treated cells [18]. 

## CLASSIFICATION OF NANOMATERIALS 

Engineered nanomaterials can be composed of many different bulk substances. Nanoparticles form a basis for many engineered nanomaterials, and are currently available in a variety of types: fullerenes (C60), carbon nanotubes (CNT), metal and metal oxide particles, polymer nanoparticles, and quantum dots are the most common. These particles are manufactured by human on the nanoscale with specific physicochemical composition and structure to exploit properties and functions associated with their dimension. Nanoscale materials can, in theory, be engineered from any chemical substance; semiconductor nanocrystals, organic dendrimers, carbon fullerenes, and carbon nanotubes represent a few of the many examples [1]. Engineered nanoparticles include particles with homogeneous composition and structure, compositionally and structurally heterogeneous particles (for instance, particles with core-shell structures), and multi functional nanoparticles (for instance, “smart” nanoparticles being developed for medical diagnostics and treatment) (Table **1**).

## FULLERENES

Fullerenes (i.e., Buckminsterfullerene or “Bucky balls”) are nanomaterials that gained attention after the first preparation of C60, a novel allotrope of carbon consisting of 60 carbon atoms joined to form a cagelike structure [19]. The unique structure of C60 facilitates absorption of light and transfer of this energy to triplet oxygen, thereby forming the highly reactive singlet oxygen state [20]. High yield of singlet oxygen with consequential generation of free radicals suggest that presence of C60 in the environment may cause oxidative damage in exposed organisms [21]. Fullerenes, characterized as antioxidants, are believed to reduce various reactive chemical species, such as free radicals, and their characteristic features have been disclosed to furnish many useful medical technologies. Despite the numerous applications of fullerenes for the biological efficacy, less is known about the toxicity of fullerenes in mammals [22]. Various water-soluble fullerenes were prepared by the use of chemical modification of hydrophobic fullerene, to widely explore the biological activities [22]. Fullerene was recognized as a free radical scavenger and water-soluble fullerenes were proved to reduce the level of intracellular peroxidation [23]. Attempts of pharmaceutical technologies with a series of water-soluble fullerenes have explored their potential as anti-HIV [24-26] and anticancer [27, 28] agents. 

From the studies cited above, we can conclude to date that fullerenes are not mutagenic under the conditions employed and no increased incidence of abnormal cells was observed *in vitro* cytogenetics test. On the other hand, fullerenes show promising pharmacotherapeutic application, so further *in vivo* studies are required.

## CARBON NANOTUBES

In a graphitic arc process that formed fullerenes from atomized carbon, Sumio Iijima in 1991 discovered multiwalled carbon nanotubes (MWCNTs), deposited at the graphite anode [29]. Shortly after, Iijima synthesized single-walled carbon nanotubes (SWCNTs) in the presence of metal catalyst [30]. These two forms have different structure: the former (SWNT) presents only one graphene layer, the latter (MWNT) presents several graphitic concentric layers. 

Carbon nanotubes are promising and unique engineered nanomaterials, and the global production of CNTs has already reached to hundreds of metric tons per year and is expanding rapidly likewise the new development of applications and manufacturers [30]. No other material has been developed that posses the size (1-20 nm in width, and many microns in length), strength, and surface chemistry properties of CNTs. The diverse utility of CNT has applied to electronic devices, polymer composites, and biochemical application such as enzymatic films, nanostructured medical devices such as tissue-engineered scaffolds, and constructs for intracellular drug/gene delivery [31]. CNT typically aggregates to various sizes ranging from a few nanometers to hundreds of micrometers in diameter in the dry state, or upon suspension in polar and non-polar solvents. CNT is involved in two separate research fields: biological/therapeutic applications such as drug delivery and gene therapy and the impact on human health and the environment. Many of the properties that make CNTs remarkable for engineering applications have also caused concerns for their biocompatibility, especially in the lungs [32]. Their length/width (aspect) ratios of > 1000, reactive surface chemistry, and poor solubility raise concerns, linked to past experience with hazardous fibers (e.g. asbestos) [32]. *In vivo* studies have revealed that intratracheally introduced CNT induce granulomas in rat [33] and in mice lungs [12, 34]. The interaction between CNTs and mammalian cells was investigated by many researcher groups. This interaction has been shown to induce anti-proliferative effects, decreased cell adhesion [17], apoptosis, necrosis [35] and oxidative stress [36]. 

## CARBON NANO-ONIONS 

Giant, nested fullerenes, generally called nano-onions (MWCNOs) [37, 38], represent a very interesting class of carbon nanoparticles. MWCNOs are usually produced by an underwater carbon-arc discharge [39, 40]. MWCNOs have been used as the components of nanocomposites for applications including solar cells, light-emitting devices and fuel-cell electrode [41]. Multilayer fullerenes (carbon nano-onions, CNOs) represent a largely unexplored carbon allotrope due to their inherent insolubility. Expectations are that the properties of these nano-onions will be unique and potentially useful, as has been the case of fullerenes and carbon nanotubes (CNTs). 

## POLYPROPYLENIMINE DENDRIMER 

Dendrimers are polymeric macromolecules that are composed of multiple perfectly-branched monomers, radially emanating from a central core. The number of branch points increases upon moving from the dendrimer core to its surface and defines dendrimer generation. The branched topology confers dendrimers with several unique properties for materials applications: compact nm-scale dendrimer structure results in high solubility and low solution viscosity. This property lends dendrimers to applications as rheology (viscosity) modifiers; dense presentation of multiple terminal groups on dendrimer surface (multivalency), with a number of surface groups increasing with dendrimer generation. The core-shell architecture can be used to encapsulate and release molecules, chemically incompatible with the environment external to the dendrimer: for example catalysts, drugs, or chromophores. Polypropylenimine (PPI) dendrimers can be used as non-viral gene-vectors; PPI have a highly branched, three-dimensional architecture with very low polydispersity and high functionality [42]. It consists of a core molecule (butylenediamine), which acts as the root from which a number of highly branched, tree-like arms originate, in an ordered and symmetric fashion [42]. Key properties in terms of the potential use of these materials in gene delivery are attributed by the high density of terminal groups. These contribute to the molecules surface characteristics, offer multiple attachment sites e.g. for conjugation of drugs or targeting moieties, and determine the molecular volume, which is important for the ability to sequester other molecules within the core of the PPI dendrimer [42].

## SILICA PARTICLES

Silica is the common name for silicon dioxide (SiO2), one of the most abundant compounds found in nature. Silica occurs in crystalline or amorphous forms. Crystalline silica is abundant in most rock types such as granites, sandstones, quartz and sands, as well as soils. Excessive exposure to crystalline silica has been linked to pulmonary diseases, such as silicosis, tuberculosis, chronic bronchitis, chronic obstructive pulmonary disease (COPD), and lung cancer [43]. Based on several toxicity studies, the International Agency for Research on Cancer (IARC) has classified crystalline silica as a Group 1 carcinogen [44]. Amorphous silica can be divided into naturally occurring or intentionally manufactured synthetic silica. Synthetic amorphous silica may be classified as wet process, pyrogenic (“thermal” or “fumed”), and chemically or physically modified silica [45]. Synthetic amorphous silica is encountered in large quantities due to occupational exposure, as it is widely used in many industries for various applications, such as fillers in the rubber industry, tire compounds, and as free-flow and anti-caking agents in powder materials; other uses are found in paints, toothpaste and cosmetics [45].

## METALLIC NANOPARTICLES

Titanium dioxide (TiO2) is a poorly soluble particulate (PSP) that has been widely used as a white pigment in the production of paints, paper, plastics, welding rod-coating material, and food colorant. Nano-sized or ultrafine TiO2 (UF-TiO2) (<100 nm) is increasingly used in other industrial products, such as cosmetics and pharmaceuticals [46]. Therefore, potential widespread exposure may occur during both manufacturing and use. Cytotoxicity induced by TiO2 was relevant to the size of particles [47]. There is an evidence that UF-TiO2 can cause inflammation, fibrosis, pulmonary damage and even DNA damage [48, 49]. UF-TiO2 might be able to enter the human stratum corneum and interact with the immune system [50, 51], since UFP can be translocated to the subepithelium space to a greater extent than the fine particles [52]. Ultrafine titanium dioxide is a nanostructure widely used in industry [53]. It occurs in three different crystalline forms (rutile, anatase and brookite). The rutile and anatase grades are of commercial importance, representing 90% and 10% of the market, respectively. Owing to its high refractive index, ultrafine titanium dioxide has light-scattering properties, hence is used in protection against UV exposure [54]. Many marketed sunscreen products contain ultrafine titanium dioxide, surface-treated with either inorganic or organic coatings, which are colorless and reflect and scatter UV more effectively than larger particles. 

Recent advances in the chemical synthesis of magnetic nanoparticles, such as cobalt nanoparticles, with controllable size and shape, are leading to new applications in a variety of fields, such as patterned media for magnetic data recording to biomedical applications like MRI contrast enhancers, DNA assays, and hyperthermia for cancer treatments [55]. This nanomaterial can be used in biological and medical applications in different forms, from the simplest, such as cobalt oxide, to complex organic compounds or biopolymers [5, 6]. Recent *in vitro* studies concerning the concurrent cytotoxicity and carcinogenic potential of Co-nano in a Balb/3T3 cell line have shown that such particles can gradually dissolve in culture medium with the generation of Co ions [56].

Amongst the array of nanomaterials, which ranged from metal and polymer nanoparticles to viral capsids developed for biological applications, gold nanoparticles (GNP) have found wide acceptance because of their stability, size controlled synthesis and relatively easy surface modification with amine and thiol groups for conjugation with DNA and proteins [57]. Colloidal gold nanoparticles have been found to strongly enhance the native signals of chemical constituents in cells. Gold nanoparticles, coated with proteins, have been used to detect conformation changes in the attached proteins *via *observation of color changes in the solution. Some reports suggested that GNP functionalized with cationic side is likely to be toxic to mammalian and bacterial cells [58]. On the contrary, Shukla *et al*. [59] and Connor *et al. *[60] have found that in spite of efficient uptake into human cells by endocytosis, GNP show little cytotoxicity. 

## RESPONSES OF *IN VIVO* SYSTEMS TO NANOMATERIALS

### Fullerenes, *In Vivo* Effects and Biomarkers 

Henry, T.B. (2007) [21] investigated the changes in survival and gene expression in larval zebrafish *Danio rerio,* after exposure to aggregates of C60 prepared by two methods: a) stirring and sonication of C60 in water (C60–water), and b) suspension of C60 in tetrahydrofurano (THF) followed by rotovaping, resuspension in water, and sparging with nitrogen gas (THF–C60). 

Survival of larval zebrafish was reduced in THF–C60 and THF–water, but not in C60–water. The greatest differences in gene expression were observed in fish exposed to THF–C60 and most (182) of these genes were similarly expressed in fish exposed to THF–water. Significant upregulation (3- to 7-fold) of genes involved in controlling oxidative damage was observed after exposure to THF–C60 and THF–water. This research is the first to link toxic effects directly to a THF degradation product (γ-butyrolactone) rather than to C60 and may explain toxicity attributed to C60 in other investigations. Two dose–response toxicity tests were conducted simultaneously with the test to evaluate changes in gene expression. The total numbers of genes in which significant changes in expression were detected relative to the control were 10 in C60–water, 217 in THF–water, and 271 in THF–C60. Up-regulation of genes with antioxidant activity (including glutathione S-transferase) in THF–water and THF–C60 treatments in the present study is consistent with the hypothesis that fishes were responding to defend against oxidative injury resulting from the exposure to oxidative chemicals. Changes in global gene expression were nearly identical in THF–water and THF–C60 treatments, and these results indicate that fish were responding to similar exposure scenarios in both treatments. THF–C60 was more toxic than THF–water, based on larval zebrafish survival and gene expression patterns. The magnitude of the change in gene expression was higher in THF–C60 for 73% of the 182 genes that were in common between THF treatments, and of the 124 genes that differed separately from the control (89 genes THF–C60, 35 genes THF–water) 72% had a higher magnitude of expression change in THF–C60. These results may be explained by either higher concentrations of γ butyrolactone (or other THF degradation products) in THF–C60, by the presence of C60, or possibly by an interaction between the C60 and γ-butyrolactone. Larval zebrafish exposed to C60 (C60–water treatment) did not die as a result of exposure, and gene expression changes relative to the control were relatively minimal. The minimal change in global gene expression, observed when zebrafish larvae were exposed to C60–water, indicates that the exposure scenario used in this investigation had only minimal effects on the fish. A final point is that changes in gene expression were investigated after a 72-hr exposure; presumably the expression of these genes (and likely other genes) will be affected differently after different exposure durations and at different zebrafish life history stages.

### Carbon Nanotubes *In Vivo* Effects and Biomarkers

There are three administrating ways: inhalation, intratracheal instillation and pharyngeal/laryngeal aspiration. In the first way, aerosolized nanoparticles are administrated under controlled condition. Physicochemical characterization of the aerosol is necessary and information regarding the particle size distribution of the aerosolized nanomaterial is of particular interest. Inhalation is the preferred method of exposure for hazard identification and to obtain dose-response data. In the intratracheal instillation, nanomaterials suspended in appropriate vehicle are administered *via *incision of trachea. It is considered as an acceptable method to evaluate the toxicity of the tested nanoparticles, but is very important to disaggregate the suspended nanomaterial. The technique to evaluate pulmonary exposure is pharyngeal or laryngeal aspiration, used to avoid contamination of food particles. This way of administration results in a uniform distribution of particles throughout the lungs. 

The study by Mitchell, L.A. (2007) [32] assessed the short term pulmonary and systemic immune response effects of MWCNTs, administrated *via *inhalation in mice. The exposure system was developed to produce CNT aerosols that simulate resuspended CNT powders that may exist in the workplace. Mouse was exposed to MWCNTs, aerosolized for 7-14 days. One of the purpose of this study was to assess gene expression after MWCNTs aerosol exposition, by real-time RT-PCR of homogenized pulmonary or spleen sample. A reverse transcription step was performed on total RNA and gene expression of interleukin 6, interleukin 10 and NAD(P)H quinine oxidoreductase 1 was assessed. They found that IL-6, IL-10, and NAD(P)H oxidoreductase 1 (NQO1) mRNA expression was not increased in the lungs, following inhalation of MWCNTs for 7 or 14 days. However, spleen mRNA levels for IL-10 and NQO1 were significantly increased with 14-day MWCNT exposure. Immune function measurements on spleen-derived cells showed suppressed, T-cell-dependent antibody response, decreased proliferation of T-cells following mitogen stimulation, and altered NK cell killing. These results were accompanied by increased NQO1 and IL-10 gene expression (indicators of oxidative stress and altered immune function, respectively) in spleen, but not in the lung.

**Fig. (1):**
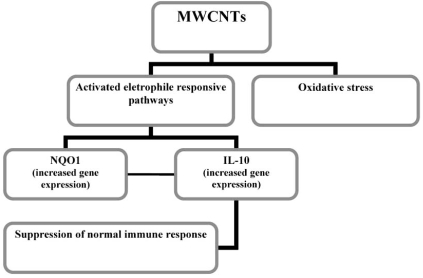
Summarizing scheme of molecular mechanism inducted by inhalation of MWCNTs in mice (Mitchell, *et al*.) [32].

IL-10 is an anti-inflammatory cytokine, principally secreted by macrophage and T cells. Its activity serves to downregulate cytokine such as IL-12, TNF-α, INF-γ and IL-1β. MWCNTs may induce oxidative stress and/or activate electrophile responsive pathways, resulting in NQO1 and simultaneous or consequent IL-10 expression; it may suppress normal immune response and increase susceptibility to infection and disease (Fig. **1**). 

SWCNT nanotubes exhibit substantial cytotoxicity *in vitro* and *in vivo* and seems to be more toxic to cells or mice/rats than multiwalled carbon nanotubes (with diameters ranging from 10 to 20 nm) [35], multiwalled carbon nano-onions (with about 30 nm in diameter) [61], fullerene (C60), [35], carbon black [12], and graphite [62]. These nanoparticles are all made of carbon atoms, but with distinct geometries and surface chemistries. Collectively, these experimental evidences reasonably suggest that the SWCNT related cytotoxicity could be attributed to their geometry (fibrous structure) and surface chemistries (e.g., electrical properties).

Chou, C.C. *et al. *(2007) [63] demonstrated that intratracheal instillation of 0.5 mg of single-walled carbon nanotubes (SWCNT) into male ICR mice, induced alveolar macrophage activation, various chronic inflammatory responses, and severe pulmonary granuloma formation. The granulomas were mainly composed of aggregates of macrophages with SWCNT particles. An investigation of the genome-wide gene expression changes in macrophages exposed to SWCNT, associated with a detailed signaling pathway analysis, ought to provide molecular insights into this type of granuloma formation. 

To address this issue, they also conducted *in vitro* experiments to investigate the effects of SWCNT on human macrophage-like cells, differentiated from a human monocytic leukemia cell line THP-1. Exposure of the human THP-1 derived macrophages to 0.05 mg/mL of SWCNT resulted in the uptake of SWCNT to form the nanoparticle-loaded macrophages, which is similar to their afore mentioned *in vivo* observations. To elucidate the SWCNT-induced cytotoxicity at the molecular level, they measured the gene expression changes in human THP-1 derived macrophages exposed to SWCNT for 24 h using Affymetrix microarrays. To analyze the complicated genome-wide gene expression data, they introduced a computational method to describe the molecular cytotoxic mechanisms induced by SWCNT exposure on the macrophages. The method weights each gene with its respective expression abundance change to evaluate and select the pathways that are most affected by transcriptional changes in genome-wide expression experiments. The data indicates that macrophages exposed to SWCNT do not undergo apoptosis, as demonstrated by the absence of active forms of Caspase-3, which is an apoptosis indicator and by a lack of DNA laddering. Therefore, the high pro-oxidant state might be continued throughout the activation of AP-1 or NF-kB. These results further indicate that AP-1, in addition to NF-kB, is activated in SWCNT-treated macrophages. This stimulation of two redox-sensitive transcription factors would not seem to be through ROS attack only, but seems to involve other signaling pathways as well. The result is a cascade of inflammatory responses involving the significant induction of a large number of proinflammatory genes and the recruitment of leukocytes. Since some target genes also serve as NF-Bk/AP-1 activators and the activation of NF-Bk/AP-1 can stimulate the macrophages themselves, the result is the recruitment of leukocytes which trigger a proinflammatory signal amplification loop for further release of inflammatory mediators. Concomitantly, the induction of various protective and survival genes by NF- kB contrasts the inflammatory responses and protects the host from excessive cellular damage. The balance between the activation of these two contradictory panels of gene expression would seem to determine the subsequent immune responses. This study shows that the SWCNT challenge endows macrophages (poor antigenpresenting cells) with an antigen-presenting function by increasing the expression level of HLA-DR, CD80, and CD40, which are able to interact with TCRs, CD28, and CD40L, respectively, on T cells, resulting in T cell activation and proliferation. The cooperative interaction between the activated macrophages and T cells then results in the formation of pulmonary granulomas.

**Fig. (2):**
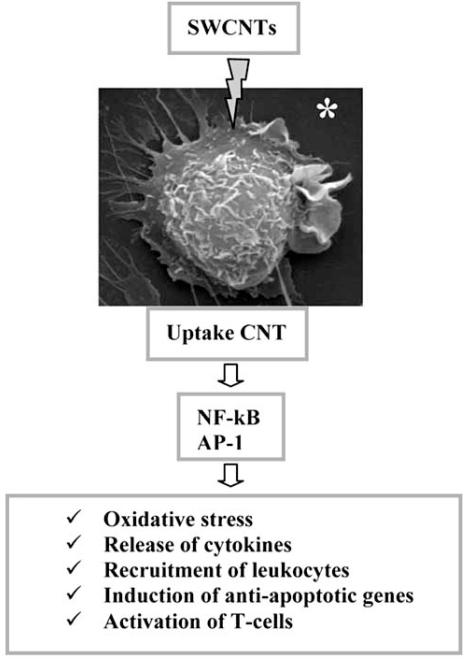
Summarizing scheme of molecular characterization of SWCNTs- induced cytotoxicity. *RAW 264.7 murine macrophage observed by SEM (Poma and Di Giorgio, for this review).

The uptake of SWCNT into the macrophages is able to activate transcription factor as nuclear factor kB and AP-1, antiapoptotic gene expression, activation of T cells, oxidative stress, and release of proinflammatory cytokines. The resulting innate and adaptive immune responses may explain the chronic pulmonary inflammation and granuloma formation *in vivo* caused by SWCNT (Fig. **2**).

## RESPONSES OF *IN VITRO* SYSTEMS TO NANOMATERIALS

### Carbon Nanotubes, *In Vitro* Effects and Biomarkers

The increasing use of nanotechnology in consumer products and medical applications underlies the importance of understanding its potential toxic effects to people and the environment. Although both fullerene and carbon nanotubes have been demonstrated to accumulate to cytotoxic levels within organs of various animal models and cell types, the molecular and cellular mechanisms for cytotoxicity of this class of nanomaterial are not yet fully apparent. 

To address this question, Ding, L., *et al. *(2005) [61] have performed whole genome expression array analysis and high content image analysis, based on phenotypic measurements on human skin fibroblast cell populations exposed to multiwalled carbon nano-onions (MWCNOs) and multiwalled carbon nanotubes (MWCNTs). Human skin fibroblasts (HSF4) and human embryonic lung fibroblasts, both untransformed cells were used to evaluate the cytotoxic and proliferative effects of carbon nanomaterials. Lung and skin cells were selected, because entry through the skin or respiratory tract is the most likely route of exposure to nanomaterials. Cells were treated with serial dilutions of MWCNO and MWCNT, and they chose doses of 0.6 and 6 mg/L for MWCNO and doses of 0.06 and 0.6 mg/L for MWCNT, so that the cells show approximately 2-fold increase in apoptosis/necrosis from the untreated baseline cells and a 50% reduction in proliferation after a treatment of 48 h at the low dose. The high doses chosen are 10 times more toxic than the low dose, so that pronounced gene expression changes can be observed to mimic the acute exposure to carbon nanomaterials. Cells were exposed for 24 or 48 h. The MWCNTs seem to be 10 times more toxic than the MWCNOs.

Treating human skin fibroblast with carbon nanomaterials induced high gene expression changes. These data indicate that, although higher doses induced a greater number of genes expression changes than low doses, there are no global dose-dependent responses to both particles. Unique genes were also induced in response to MWCNO or MWCNT and more genes demonstrated changes in levels of expression at the lower concentration of MWCNO. Interestingly, it is the dosage of carbon nanomaterials that appears to have the greatest influence on gene expression changes common between MWCNOs and MWCNTs, not the specific nanomaterial. In summary, combined with the result from functional analysis, this study clearly showed that at high dosage, carbon particles can seriously influence the cellular functions of maintenance, growth and differentiation. Of these two nanomaterials, MWCNTs appear to induce more stress on the cells than MWCNOs. Data suggest that there is a qualitative difference in response to low dose and high dose treatment of carbon particles in human skin fibroblasts. Carbon tubes at high dose induced innate immune responses, whereas carbon onions did not. This indicates that cells answer differently according to the structures of nanomaterials. These data also suggest that carbon atoms released from nanomaterials may participate in cell metabolic pathways. It is evident from this study that carbon nanomaterials have a toxic effect on lungs and skin cells.

MWCNO and MWCNT exposure activates genes that are involved in cellular transport, metabolism, cell cycle regulation, and stress response. MWCNTs induced genes are indicative of a strong immune and inflammatory response within skin fibroblasts, while MWCNO changes are concentrated in the genes, induced in response to external stimuli. Results of Promoter analysis of microarray demonstrate that interferon and p38/ERK-MAPK cascades are critical pathway components in the induced signal transduction, contributing to the more adverse effects observed upon exposure to MWCNTs as compared to MWCNOs. 

It is important to underline that carbon nanotubes are now becoming an important material for use in day to day life, because of their unique physical properties. The toxicological impact of these materials has not yet been studied in detail, thereby limiting their use. From *in vitro *observations, it seems likely that carbon nanoparticles can interfere with the NF-kB pathway, because of their ability to induce TNF-alpha and oxidative stress [36, 64].

The work of Manna, S.K. (2005) [16] evaluated the effect of carbon nanotubes in HaCaT cells to determine growth-inhibiting potential and oxidative stress. Moreover, they want to investigate the role of NF-kB in the cytotoxic mechanism induced by SWCNTs. They show the involvement of NF-kB in single-walled carbon nanotubes (SWCNTs) induced toxicity in HaCaT cells. To study the induction of oxidative stress induced by SWCNT particles, HaCaT cells were exposed to different concentrations of SWCNT particles and generation of reactive oxygen species (ROS) was monitored through increases in fluorescence intensity of dichlorofluorescin (DCF). The results show a dose-dependent increased generation of ROS by SWCNT particles in HaCaT cells. A significant increase in ROS is shown at concentrations ranging from 1 to 10 μg/mL. To further assess the extent of damage as a result of oxidative burst by SWCNT particles, cell viability was determined after exposing the cells to various concentrations of SWCNT particles. This group has previously shown that oxidative stress can activate NF-kB, stress-activated kinases, and such activation could result in cell death by either apoptosis or necrosis [65, 66]. In the next series of experiments the activation of NF-kB was investigated by exposing HaCaT cells to various concentrations of SWCNT particles for 12 h and performing enzyme mobility shift assays (EMSA). SWCNT particles could activate NF-kB as a result of increased oxidative stress. For the first time this group suggested that SWCNT particles are capable of inducing NF-kB in a dose-dependent manner, in HaCaT cells. To further examine the binding specificity, EMSA was carried out in the presence of antibodies of p50 and p65 proteins; p50 and p65 proteins translocate the nucleus and bind to the DNA forming the active component of the NF-kB complex [67]. These observations indicate that HaCaT cells exposed to SWCNT particles activate NF-kB and strongly suggest a role of NF-kB in the process of cytotoxicity. NF-kB is an important transcription factor and has been shown to participate in cell death and in inflammatory responses [68]. The hypothesis is that NF-kB activation by SWCNT particles could lead to the binding of the activated complex to the promoter sequences, and thus aid in transcription. These results reveal that there might be a specific signaling mechanism being triggered by SWCNT particles and that the down stream effect of this pathway leads to cell death. One of the events that is caused by the treatment with SWCNT particles may be the activation of the NF-kB. Since NF-kB is involved with cytokine-mediated signaling, it could be speculated that SWCNT particles can interfere with or mimic cytokine signaling which might be the cause of the inflammation.

In another study Chui, D. and his group (2004) [17] investigated the influence of single-walled carbon nanotubes (SWCNTs) on human HEK293 cells (human embryo kidney cells), with the aim of exploring SWCNTs biocompatibility. Results showed that SWCNTs can inhibit HEK293 cell proliferation, decrease cell adhesive ability in a dose- and time-dependent manner, active responses to SWCNTs such as secretion of some 20–30 kd proteins to wrap SWCNTs, aggregation of cells attached by SWCNTs and formation of nodular structures. Moreover, SWCNTs can induce down-regulation expression of adhesion-associated proteins such as laminin, fibronectin, cadherin, FAK and collagen IV. Cell cycle analysis showed that 25 μg/ml SWCNTs in a medium, induced G1 arrest and cell apoptosis, in HEK293 cells. Cell cycle, cell apoptosis and signal transduction gene expression were investigated by Biochip analysis. Cells were cultured for 48h with SWCNTs (25μg/ml) or without and gene expression was analyzed by oligonucleotide microarray. The analysis showed that cells were arrested in the G1 phase and this arrest was accompanied by a dramatic decrease in the number of cells in the S phase, with Rb/p53 as the main apoptosis pathway induced by SWCNTs. Data showed that SWCNTs can induce up-regulation expression of cell cycle-associated genes such as p16, bax, p57, hrk, cdc42 and cdc37, down-regulation expression of cell cycle genes such as cdk2, cdk4, cdk6 and cyclin D3, and down regulation expression of signal transduction-associated genes such as mad2, jak1, ttk, pcdha9 and erk. In SWCNTs-treated HEK293 cells, accumulated p16 protein may bind to and inhibit the kinase activity of cdk2, cdk4, and cdk6, hence prevent the cells from entering into the S phase and subsequently arrest the cell cycle in the G1 phase. Their observations show that SWCNTs can induce HEK293 cell apoptosis, which were characterized by morphological changes, chromatin condensation, and internucleosomal DNA fragmentation, accompanied by up-regulation expression of apoptosis-associated genes such as p16, bax, hrk, bak1, p57, FGFR2, TGF beta receptor 1 and TNFAIP2 genes and downregulation expression of cell cycle-associated genes such as cyclin D1, cdk2, cdk4, and cdk6 as compared to normal HEK293 cells. Data showed that the expression of bax and bcl-Xs were upregulated in SWCNTs-treated HEK293 cells and the bcl-2 family is involved in the cell apoptosis induced by SWCNTs. Chui, D. and his group proposed a possible model of interaction between singlewalled carbon nanotubes and HEK293 cells: SWCNTs attached to the surface of HEK293 cells, provides stimuli signal to the cells. The signal is transduced inside the cells and the nucleus, leading to downregulation of adhesion-associated genes and corresponding adhesive proteins, resulting in decrease cell adhesion and causing cells to detach, float and shrink in size. At the same time, SWCNTs induce up-regulation of apoptosis-associated genes such as p16, Rb, and p53 and cause HEK293 cells arrest in the G1 phase, finally resulting in apoptosis. During this period, HEK293 cells make active responses of self-protection to SWCNTs, secrete some small proteins into the medium to wrap SWCNTs into nodular structures, which isolate the cells attached by SWCNTs from the remaining cell mass.

Sarkar, S. (2007) [69] explored the effects of single-walled carbon nanotubes on the stress gene in human BJ Foreskin cells. The results show induction of oxidative stress in SWCNTs (6μg/ml) treated cells and increase in stress responsive genes. The genes include inducible genes like HMOX1, HMOX2, and Cyp 1B1. In addition, they investigate increase for four genes by SWCNTs, namely ATM, CCNC, DNAJB4, and GADD45A, by RT-PCR. In order to investigate gene expression, BJ Foreskin cells were treated with 6μg/ml for 24h and than stress and toxicity array was performed. The gene expression changes were validated by RT-PCR. Oxidative stress can affect multiple signaling pathways, which can be transcriptional activation to inactivation genes, phosphorylation, and dephosphorylation of protein [70, 71]. Data show that SWCNTs induce significant increase in ROS in cells, thereby these findings allow speculating considerable changes in stress response genes. 28 genes, involved in apoptosis, xenobiotic metabolism, DNA repair, oxidative stress and production of chemokine, showed significant increase (ratio ranging from 1.5 to 3) in treated cells as compared to control. Therefore, the global trend in the gene expression was an increased response to the stress induced by SWCNTs. The gene that showed the higher increase was HMOX2 followed by HMOX1, an inducible gene, indicating that inducible genes can be activated under the stress of this material. Heme oxigenases are microsomal enzymes that catalyze the oxidative cleavage of the porphyirn ring to generate biliverdin, free heme iron, and carbon monoxide (CO) [72]. HMOX1 contributes to physiological functions such as anti-oxidative, anti-inflammatory, anti-proliferative, and anti-apoptotic effects. This gene is an inducible isoform, evolutionary conserved and ubiquitously distributed in tissues. SWCNTs induce stress in the cells, and thus induction of HMOX1 may be protective adaptation to the stress. In the same manner increased expression of HMOX2 and catalase suggest that cells counteract the oxidative stress. ERCC4, a human gene involved in the nucleotide excision repair (NER) pathway, was also increased after SWCNTs treatment. Apoptosis that results after the treatment with SWCNTs might result in the induction of ERCC4, as a counter measure, to protect the cells from DNA damage. They even found an increase in TP53 and Caspase 8 that are genes involved in apoptosis. Moreover the genes involved in inflammatory response (macrophage migration inhibitor factor, MIF; IL18) showed significant alteration in treated cells. They validated few of the genes altered by the treatment, with SWCNTs, with RT-PCR. These genes were: Ataxia telangiectasia mutated (ATM), Cyclina C (CCNC), DnaJ (Hsp40) homolog (DNAJB4) and Growth arrest, and DNA-damage-inducible alpha (GADD45A). SWCNTs increase ATM (protein kinase involved in DNA double strand break signaling) significantly and this possibly indicates that the cell cycle might be stalled and DNA damage may also be speculated due to ROS generation. GADD45A is induced under genotoxic effects and its increase after the treatment with SWCNTs leads cells toward apoptosis. In summary, they conclude that SWCNTs treatment induce significant ROS and influence stress response genes. The induction of ROS can induce several genes as a response to counteract the physiological changes in the cells. The genes affected by SWCNTs treatment include genes involved in oxidative stress, apoptosis, DNA repairs genes, genes encoding for chaperon proteins, and cytochrome p450 family. The changes in gene expression reveal that these particles are capable of manipulating signal transduction pathways through alteration in stress related genes. In the previous cited works, all the *in vitro* treatments demonstrated dose-dependent response generation (range from 1 to 10 μg/ml, realistic when compared to actual occupational exposures).

### Polypropylenimine Dendrimers, *In Vitro* Effects and Biomarkers

Recently, polypropylenimine (PPI) dendrimers have emerged as attractive cationic vectors for the delivery of nucleic acids [73]. The lower-generation of PPI/diamino-butane (DAB) dendrimers (generation 2 (DAB-8) and generation 3 (DAB 16) have been reported to be affective gene-transfer [74], as well as being effective delivery system for antisense oligonucleotides (ODNs) in human epithelial cells [73]. In their report Omidi and his group (2005) [75] tried to assess the impact of cationic PPI-dendrimers on global gene expression, and studied the toxicogenomics of the two lower generation of dendrimers (DAB 8 and DAB 16) in human epidermoid A431 cells and in human lung epithelial A549 cells. They showed for the first time that PPI-dendrimers can intrinsically alter the expression of many endogenous genes that are dependent on the dendrimer generation and cell type.

They isolated RNA from dendrimer alone and PPI-DNA treated cells and than carried out a set of assay to investigate gene expression (Aminoallyl-dUTP labelled cDNA microarray, Hybridization of Cy-dye coupled aa-DNA, analysis of cDNA microarrays and semi-quantitative RT-PCR) and DNA damage (COMET assay). To assess the appropriate concentration of polymer, they performed MTT assay on treated cells (PPI-DNA treated cells appear more viable than dendrimers alone treated ones). They found that PPI-dendrimers, separated from their capability as transfection reagents, can intrinsically alter the expression of many endogenous genes that could potentially lead them to exert multiple biological effects on the cells. In addition, this toxicogenomics study shows that PPI treatment, at concentration routinely used for transfection of nucleic acids, on cells leads to alteration of genes involved in apoptosis and cytokine signaling. 

### Amorphous Silica Particles, *In Vitro* Effects and Biomarkers 

Cho, W.S. (2007) [76] evaluate the pulmonary effects and inflammatory mechanisms of ultrafine amorphous silica particles (UFASs), intratracheally administered in A/J mice. UFASs suspension was prepared in PBS and administered to A/J mice at doses of 0, 2, 10 and 50 mg/kg. The histopathological examination revealed that UFASs induce severe inflammation, with neutrophils, at an early stage and chronic granulomatous inflammation at the later stage. The mRNA and protein levels of IL-1β, IL-8, TNF-α, MCP-1, and MIP-2 in lung tissues were significantly increased during the early stages (24 h), but there were a decrease after no changes after weeks 1 week after instillation (TNF-α) or 4 weeks (IL-1β, IL-6, IL-8, MCP-1 and MIP-2). Instillation of UFASs-induced transient, but very severe lung inflammation. Therefore, the cytokines (IL-1β, IL-6, IL-8 and TNF-α) and chemokines (MCP-1 and MIP-2) play important roles in the inflammation induced by the intratracheal instillation of UFASs. The response was transient for amorphous silica, but sustained with post-crystalline silica exposure [77, 78]. These findings have been explained by several studies; that is, ultrafine particles (diameter<0.1μm) may translocate from the site of deposition in the lungs to extrapulmonary organs through the systemic circulation [11, 79, 80], which might result in the rapid elimination of lung inflammation and injury. However, during the early event after instillation, the severity of injury due to ultrafine particles was more severe than that of fine particles [81, 82]. IL-1β, regarded as a principle mediator of inflammation, is involved in a variety of cellular activities, including cell proliferation, differentiation, and apoptosis. Also, this gene triggers the recruitment of chemokines, which play roles in the inflammation process [83]. IL-6, a pleiotropic cytokine with multiple activities, is produced by many cell types, such as T lymphocytes, macrophages, monocytes, endothelial cells, and fibroblasts [84]. Also, it has been reported that TNF-β is responsible for the induction of chemokines in the lungs in response to silica, and that the expression of chemokines is modulated by the presence of TNF-β. MCP-1, produced by monocytes/macrophages and fibroblasts, as well as by epithelial and endothelial cells, is considered one of the potent chemotactic chemokine for monocytes, activated lymphocytes, eosinophils, and neutrophils [85, 86]. MIP-2, a member of the alpha (C-X-C) subfamily of chemokines, induces the migration of neutrophils to sites of inflammation [87], is produced by macrophages, neutrophils, fibroblasts, epithelial cells and activated astrocytes, and increases due to the direct oxidant stress induced by silica exposure [88]. In summary, the intratracheal instillation of UFASs to mice was found to up-regulate cytokines (IL-1β, IL-6, IL-8, and TNF-α) and chemokines (MCP-1 and MIP-2) early after their instillation. Furthermore, these genes and inflammatory lesions were transient and rapidly recovered to near the control levels from inflammation.

### Metallic Particles, *In Vitro* Effects and Biomarkers

To examine the possible neurotoxicity of TiO2, nerve cells critical to the pathophysiology of neurodegeneration (i.e., microglia, neurons) were exposed to a commercially available nanomaterial, Degussa P25 [89]. This material is an uncoated photo-active, largely anatase form of nanosized TiO2, not to be confused with the nonphotoactive nanomaterial currently used in sun blocks and cosmetics. P25 is a widely distributed material used for water treatment, self-cleaning windows and antimicrobial coatings and paints. The BV2 microglia is an immortalized mouse cell line. Its biochemical, morphological and genomic response to P25 exposure was examined. BV2 microglia were exposed to P25 (20 ppm) for 3 hr and total RNA was extracted. Large-scale gene analysis was performed by Expression Analysis, using the Affymetrix Mouse Genome 430 2.0 GeneChip oligonucleotide array that measures approximately 39,000 transcripts. Data analysis indicated that P25 up-regulated genes were clustered around signaling pathways involved with B-cell receptor (gene transcription in the immune response), the death receptor (tumor necrosis factor receptor family; apoptotic initiating pathways; caspase activation), apoptosis, calcium, and inflammation [(nuclear factor (NF)-kB)]. Several up-regulated cell cycling and maintenance pathways included neuregulin and ERK/MAPK (extracellular signal-regulated kinase/mitogen activated protein kinase) receptor (growth factors for cell proliferation, differentiation, migration, survival, and fate). Toxicity analysis indicated a strong pathway association with pathways associated with inflammation (NF-kB), cell cycling, oxidative stress (peroxisomes) and pro-apoptotic activities. P25’s down-regulated genes were associated with adaptive change (e.g., B-cell receptor, ERK/MAPK) and energy production (glycolysis, gluconeogenesis, oxidative phosphorylation). The present data indicates that Degussa P25 stimulates BV2 microglia to release ROS and affects genomic pathways associated with cell cycling, inflammation, apoptosis, and mitochondrial bioenergetics. In summary, this study describes the *in vitro* neurotoxicity of a widely used nanomaterial, P25. This material appears to be non-toxic to isolated N27 neurons, but stimulates BV2 microglia to produce ROS and damages OS-sensitive neurons in cultures of brain striatum.

Papis, E. (2007) [90] focused attention on mRNA expression in a BALB3T3 clone A31-1-1 cell line that was not exposed and exposed for 72 h to 1 μM of cobalt microparticles (Co-μ), nanoparticles (Co-nano), and ions at the inhibitory concentration of 20% of the control (IC20) for72 h. The expression of some mRNAs was found to be modified by the treatment; in particular, they found some interesting genes involved in the inflammatory response, such as the interferon-activated gene 203. The expression of RAB GTPase mRNA was modified by the treatment. RAB GTPase is involved in the secondary cellular response and belongs to a family of GTPase-activating proteins, or GAPs, these are regulatory proteins whose members can bind to activated G proteins and stimulate their GTPase activity, thus terminating the signaling event. Among the modified mRNA, only the band 4GP49 proved to be down regulated after exposure to Co-nano. By BLASTn analysis, they found that this band corresponds to degenerative spermatocyte homolog1. This gene encodes for a member of the membrane fatty acid desaturase family, which is responsible for inserting double bonds into specific positions in fatty acids. Overexpression of this gene inhibited biosynthesis of the EGF receptor, suggesting a possible role of a fatty acid desaturase in regulating biosynthetic processing of the EGF receptor. In summary, in this study Papis and his group obtained 10 differently expressed sequences. These genes represent candidate biomarkers capable of indicating specific cellular effects after Co-nano exposure. In addition, their results show that treatment with Co-nano somehow activates cellular pathways of defense and repair mechanisms. They cannot exclude that the differences found in the expression of some RNA are entirely due to the size and not to the cobalt itself. Even though these results do not permit conclusions to be drawn about the molecular mechanisms involved, they do suggest that the treatment with Co-nano somehow activates cellular pathways of defense and repair.

Khan, J.A. (2007) [91] investigated the transcriptional profile of HeLa cells in the presence of gold nano-particles (GNPs), along with cytotoxicity and stress-specific assay to assess the effects of GNPs. In this study 18 nm GNPs were administered to HeLa cells and then MTT assay, uptake studies, and gene expression profiling were performed. The ability of nanoparticles to enter the cells is an important property, since it can be used to deliver DNA or drugs. GNP, in spite of being internalized by cells, did not trigger significant cytotoxicity. The presence of xenobiotic agents or changes in the intracellular or extracellular environment usually trigger specific pathways of stress response, such as induction of chaperonines, ribosomal protein synthesis, activation of stress-specific kinases, cytochrome P450 expression and glutathione transferase activity. An effective stress response mounted by cells is directed towards preventing cell death. Cell death is therefore the extreme end point observed, when natural cellular defense has been ineffective in countering stress. Khan, J.A. generated transcriptional profile of HeLa cells incubated with 18 nm GNP for 6h, and compared these with untreated control cells by using both expressed sequence tag (EST) arrays that carried probes for 19000 human genes and high-density oligonucleotide arrays, with probes against 47000 transcripts and variants. They compared the genes that were affected by the exposure to GNPs with carbon particles affected ones. Exposure to carbon nanotubes induce the expression of genes involved in the pathways like immune response, transport, cell cycle regulation, apoptosis and external stimuli sensing [61]. However, GNP did not induce any significant changes in the expression level of genes involved in these pathways. The transcriptional profile of HeLa cells exposed to GNP did not include known stress response pathways. In summary, this study shows that there were no gross changes in gene expression patterns after uptake of gold nanoparticles into this human cell line.

## CONCLUSION

Application fields of engineered nanomaterials are various and include industrial, physical, electric ones or biological and medical application fields. Production of nanomaterials is greatly developing implying their diffusion in the environment both as nano-particulate and in many industrial products such as cosmetics or drug. In this case nanomaterials can enter in the organism *via *inhalation, ingestion or epidermal contact.

**Fig. (3):**
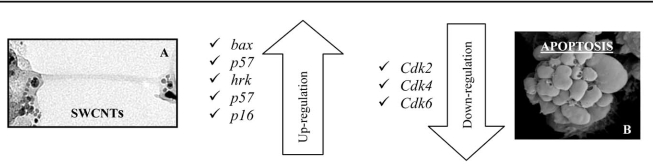
Effects of SWCNTs treatment on cell cycle genes. Affected gene expression lead cells to apoptosis. A) SWCNT in cell media observed by TEM; B) RAW 264.7 murine macrophage observed by SEM (Poma and Di Giorgio, for this review).

Several studies have reported that inhaled or injected nanosized particles enter in the systemic circulation and migrate to various organs and tissues [92, 93], where they could accumulate and damage organ systems that are sensitive to oxidative stress (OS). The brain is one such organ, being highly vulnerable to OS because of its energy demands, low levels of endogenous scavengers (e.g., vitamin C, catalase, superoxide dismutase) and high cellular concentration of OS targets (i.e., lipids, nucleic acids, and proteins) [89]. In the brain, OS damage is mediated by the microglia, a macrophage-like, phagocytic cell that is normally inactive unless confronted by potentially damaging xenobiotics. Their immediate and characteristic response (i.e., “oxidative burst”) to foreign stimuli involves cytoplasmic engulfment (i.e., phagocytosis), an increase in metabolic activity, and a change in cell shape, size and proliferation [94]. The excess O2^–^ arising from the oxidative burst can diffuse from the microglial plasma membrane and damage the proteins, lipids, and DNA of neighboring cells, especially neurons. It is evident that nanomaterials may affect cell signaling through interaction with plasma membrane. They can also be transported within a cell *via *endocytosis and interfere with normal cellular functions by interacting with intracellular molecular target. Nanomaterials may have a complex structure and display multiple functional groups at the surface charged or even chemically reactive [95]. Polymodal receptors located in the cellular membrane of microglia and macrophages (e.g., TRPV1, Mac-1) are sensitive to protons (i.e., charge) or repeating patterns of charge [96] like those found on crystalline metal oxide nanoparticles. The activation of these receptors triggers various signal transduction pathways that determine the cell’s ultimate fate. Nanoparticles are small and have a wide surface area, and thus make them very harmful for human health. Moreover, the interaction of nanoparticles with cell membrane and the transport into cells are poorly understood [95]. Data showed in this review, clearly underlines that engineered nanoparticles influenced different signaling pathways. Nanoparticles treatment both in *in vitro* and *in vivo* systems mainly affect the expression of genes involved in inflammatory response, apoptosis and oxidative stress (Tables **2** and **3**). These activated pathways allow organism to defend from injury caused by nanoparticles treatment. Interesting data have been shown in the study of Mori [22], where they investigated toxicity of C60 in an *in vivo* system (larval Zebrafish). They found an up-regulation of genes correlated with oxidative damage, but they observed that the use of THF (tethaydrofuran) used as vehicle resulted in the generation of degradation products (γ-butyrolactone) and toxic effects. On the contrary, data on CNTs clearly shows that these nanoparticles interact with cell membrane [17] and cause up-regulation of genes involved in oxidative stress (NQO1, [32]; HMOX1, HMOX2, [69]), apoptosis (p16, Rb, p53, Chui, D. [17]; NFk-B, [16]), and inflammatory response (IL-10, [32]). In addition, the toxicogenomics studies show that nanomaterials treatments on the cells lead to alteration of genes involved in apoptosis and cytokine signaling. SWCNTs (to date the more extensive study nanomaterials) can induce cell apoptosis, which is characterized by morphological changes, chromatin condensation and internucleosomal DNA fragmentation, accompanied by up-regulation expression of apoptosis-associated genes such as p16, bax, hrk, bak1, p57, FGFR2, TGF beta receptor 1 and TNFAIP2 genes and downregulation expression of cell cycle-associated genes such as cyclin D1, cdk2, cdk4, and cdk6 as compared to normal cells (Fig. **3**). In the same way, silica particles activate cytokine signaling (IL-1β, IL-6, IL-8, TNF-α, [76]) in mice intratracheally instilled, but Cho *et al. *[76] observed that the effect is transient because silica nanoparticles are rapidly translocated from lungs to extrapulmonary organs. This review suggests that cells answer differently according to the chemical structures and physical characteristics of nanomaterials. In general, the toxicity studies collectively show that all the nanomaterials tested, regardless of the method of synthesis, could induce cellular effects. However, we here emphasize that the presence of impurities might affect the severity of lesions. We remember that depending on the manufacturing processes and postsynthetic purification, i.e. the non nanotube carbon and the metal residues in a CNT product can vary greatly, so an equal amount of these two products will be expected to produce different degrees of toxic results to treated animals or cells. We also underline the role of agglomeration in the cytotoxic effect of nanomaterial; we remember that because of their geometry and hydrophobic surface, CNTs have a tendency to form agglomerates with a bundle-like form. In summary, critical features that seem to determine CNT toxicity are the presence of carbonaceous material and the degree of CNT dispersion, which could be checked in any treatment. 

Toxicogenomics could significantly contribute to the explanation of modes of nanomaterials action in parallel to traditional approaches and other –omic technologies (i.e. proteomics and metabonomics). The measurement of gene-expression levels upon exposure to nanomaterials can not only provide information about the mechanism of action of nanotoxicants, but also a “genetic fingerprinting” from the pattern of gene expression changes it elicits *in vitro* and *in vivo*. We conclude that new toxicogenomic methods are expected to have the power and potential to change nanotoxicology.

## Figures and Tables

**Table 1.** Cited Nanomaterials with Characteristics, Applications and their Effects on *In Vitro* and *In Vivo* System

Particle	Characteristics	Application Field	Toxicity Data
Fullerene (C60)	Spherical molecule with 60 carbon atoms arranged in a cagelike structure	Medical technologies (free radical scavenger, anti-HIV)	Oxidative damage [21]
Carbon nanotube (CNT)	Single-walled carbon nanotubes: one grapheme layer; Multi-walled carbon nanotubes (MWCNTs):several concentric grapheme layer	Electronic device, enzymatic films, tissue-engineered scaffolds, drug/gene delivery	Granulomas in rat [33] and in mice lungs [12, 34]. Anti-proliferative effects, decreased cell adhesion [17], apoptosis, necrosis [35] and oxidative stress [36]
Carbon nano-onion (CNO)	Giant nested fullerene	Solar cells, light emitting-devices, fuel-cell electrode	Impact cellular functions such as maintenance, growth and differentiation [61]
Polypropylenimine dendrimer (PPI)	Polymeric molecules composed of multiple branched monomers radially emanating from a central core	Non-viral vector for nucleic acids and drugs delivery	Inadvertent gene expression change and apoptosis in human carcinomas cells [75]
Silica	SiO2 ; crystalline or amorphous form	fillers in the rubber industry, tire compounds, paints, toothpaste cosmetics	Pulmonary diseases, such as silicosis, tuberculosis, chronic bronchitis, chronic obstructive pulmonary disease (COPD) and lung cancer [43]; activation of inflammatory response in mice [76]
Titanium dioxide	TiO2; poorly soluble particulate	White pigment in paints, paper, plastic, food colorant; cosmetics and pharmaceuticals (ultrafine TiO2)	Inflammation, fibrosis, pulmonary damage and even DNA damage [48, 49]; enter the human stratum corneum and interact with the immune system [50, 51]
Metallic cobalt	CoCl2	magnetic data recording, biomedical applications, DNA assays, and hyperthermia for cancer treatments	Alteration of mRNAs synthesis [90]
Gold nanoparticles (GNP)	Suspension of sub-micrometer-sized particles of gold in a fluid. Variety of shape: spheres, rods, cubes, and caps are some of the more frequently observed ones	conjugation with DNA and proteins (detect conformation changes in the attached proteins)	Contrasting results about toxicity from different studies are toxic to mammalian and bacterial cells [58]; little cytotoxicity [59, 60]

**Table 2.** Genes Affected by CNTs Treatment (*Up-Regulation)

Particles	Gene	Pathway	System	Author
MWCNTs	IL-10 IL-6 NQO1	Cytokine signaling Electrophile response	Mice (C57BL/6)	Mitchell *et al.* [32]
SWCNTs	AP1 NFk-B HLA-DR CD80 CD40	Redox-sensitive transcription factor Macrophage antigen-presenting function	Mice (ICR)	Chou *et al. * [63]
SWCNTs	NFk-B	Cell death and inflammatory response	Human keratinocytes (HaCaT)	Manna *et al. * [16]
SWCNTs	p16* bax p57 hrk cdc42 cdc37 cdk2 cdk4 cdk6 cyclinD3 mad2 jak1 ttk pcdha9 erk p16* baxhrk bak1 p57 FGFR2 TGF beta receptor TNFAIP2	Cell cycle Signal transduction Apoptosis	Human embryo kidney cells (HEK293)	Chui *et al.* [17]
SWCNTs	HMO1 HMO2 ERCC4 TP53 Caspase8 ATM GADD45A	Anti-oxidative, anti-inflammatory functions Protection from DNA damage Apoptosis Cell cycle Induced under genotoxic effect	Human BJ Forskin cells	Sarkar *et al.* [69]

**Table 3.** Genes Affected by Nanomaterials Treatment

Particles	Gene	Pathway	System	Author
PPI	IL-9 (gp96)-1 Cyclin h Cyclin a1	Inflammatory response Tumor rejection antigen Cell cycle	Human epidermoid cell (A431) Human lung epithelial cells (A459)	Omidi *et al.* [75]
Amorphous silica particles	IL-1β IL-6 IL-8 TNF-α MCP-1 MIP-2	Cytokine signaling Chemokine signaling	A/J mice	Cho *et al.* [75]
TIO_2_	B-cell receptor Death receptor NFk-B	Immune response Apoptosis	BV2 microglia (immortalized mouse cell line)	Long *et al.* [89]
CoCl_2_	Interferon-activate gene 203 RAB GTPase degenerative Spermatocyte homolog 1	Inflammatory response Secondary cellular response Involved in sintesis of fatty acids	Mouse fibroblast (Balb/3T3)	Papis *et al.* [90]
